# The Effectiveness of mHealth Interventions Targeting Parents and Youth in Human Papillomavirus Vaccination: Systematic Review

**DOI:** 10.2196/47334

**Published:** 2023-11-21

**Authors:** Lihong Ou, Angela Chia-Chen Chen, Ashish Amresh

**Affiliations:** 1 Edson College of Nursing and Health Innovation Arizona State University Phoenix, AZ United States; 2 College of Nursing Michigan State University East Lansing, MI United States; 3 College of Engineering, Informatics, and Applied Sciences Northern Arizona University Flagstaff, AZ United States

**Keywords:** human papillomavirus, mobile health, mHealth, parents, systematic review, vaccination, youth, mobile phone

## Abstract

**Background:**

The prevalence of human papillomavirus (HPV) and its related cancers is a major global concern. In the United States, routine HPV vaccination is recommended for youth aged 11 or 12 years. Despite HPV being the most common sexually transmitted infection and the vaccine’s proven efficacy, the vaccination rate among US youth remains below the recommended 80% completion rate. Mobile health (mHealth) interventions have demonstrated promise in improving health. Examining and synthesizing the current evidence about the impact of mHealth interventions on vaccination coverage in youth and intervention characteristics could guide future mHealth interventions aimed at mitigating the vaccination gap and disease burden.

**Objective:**

This study aims to conduct a systematic review to assess the effectiveness of mHealth interventions on parental intent to vaccinate youth against HPV and youth’s vaccine uptake.

**Methods:**

We searched empirical papers through databases including Google Scholar, PubMed, CINAHL, PsycINFO, and Cochrane Library. The inclusion criteria were the following: (1) published between January 2011 and December 2022; (2) using mHealth aimed to improve HPV vaccination rate; (3) targeted unvaccinated youth or their parents; and (4) measured HPV-related knowledge, vaccination intention, or vaccine uptake. Overall, 3 researchers screened and appraised the quality of the eligible papers using the Melnyk Levels of Evidence and the Cochrane Grading of Recommendations Assessment, Development, and Evaluation methodology. Disagreements in search results and result interpretation were resolved through consensus.

**Results:**

Overall, 17 studies that met the inclusion criteria were included in the final review. Most studies were conducted in the United States (14/17, 82%), used a randomized controlled trial design (12/17, 71%), and adopted behavior change theories or a culture-centric approach (10/17, 59%). mHealth interventions included SMS text message reminders, motivational SMS text messages, computer-tailored or tablet-tailored interventions, smartphone apps, web-based tailored interventions, social media (Facebook) campaigns, digital videos, and digital storytelling interventions. Approximately 88% (15/17) of the mHealth interventions demonstrated positive effects on knowledge, intention, or behaviors related to HPV vaccination. Overall, 12% (2/17) reported limited or no intervention impact on vaccine uptake or vaccine series completion. Effective vaccine uptake was commonly seen in interventions based on behavior change theories and those that provided culturally relevant information.

**Conclusions:**

This systematic review identified the impact of mHealth interventions among unvaccinated youth and their parents, which showed improvement in HPV-related knowledge, vaccination intention, or vaccine initiation. The interventions that incorporated theories and culture-centric approaches revealed the most promising results. Although these outcomes are encouraging, future studies are needed to investigate factors associated with the success of interventions using SMS text messaging or social media. More studies are also needed for a better understanding of the intervention elements that boost the responses of age-specific and ethnicity-specific populations.

## Introduction

### Background

Human papillomavirus (HPV) is the most common sexually transmitted infection in the United States and globally. In the United States, the overall prevalence of HPV infection was estimated at approximately 40%, with the incidence of disease-associated HPV infection of 6.9 million and 6.1 million among men and women, respectively [1]. Each year, approximately 13 million individuals including adolescents were found infected with the virus [2]. Individuals who are infected with the virus could be asymptomatic, which makes the diagnosis of the disease difficult [3]. With the potential to cause genital warts and cancers in the cervix, penis, anus, and oropharynx, the considerable disease burden attributable to HPV infections remains as a global concern.

The HPV vaccine could reduce the risk of developing HPV-related infections and cancers, making it a critical tool for protecting individuals from these potentially life-threatening diseases. The prevention and control of the disease could be more difficult to manage owing to the low vaccination rate [4-6]. Since the introduction of the first HPV vaccine in 2006, the Advisory Committee on Immunization Practices has recommended starting the vaccination at age 9, with routine vaccination by age 11 or 12, to ensure protection before potential virus exposure. However, despite these guidelines, the current vaccination rate among US youth stands at only 58.6%—lagging behind the 80% target set by the Healthy People 2030 initiative, which aims to promote health and well-being of well-being of individuals, organizations, and communities across the United States [7,8].

### Role of Mobile Health

Mobile health (mHealth), as part of digital health interventions, uses mobile, wireless technologies to deliver information [9]. Previous studies have found this approach to be promising for closing gaps in health systems by facilitating the successful delivery of various health services or interventions and improving the quality of care. For example, mHealth apps facilitated point-of-care diagnostics, tracked crucial events, and contributed to data collection and decision-making among health care providers [9]. Using mHealth was also found to be beneficial in supporting patients with cancer throughout their disease journey, improving their quality of life, and promoting overall patient well-being [10]. Nevertheless, previous studies have shown the inconsistent effectiveness of mHealth in promoting youth vaccination. For instance, a review paper examining the effectiveness of mHealth solutions in facilitating vaccination campaigns for unvaccinated children living in low-income and middle-income countries has suggested a promising impact on vaccine uptake [11]. However, an mHealth intervention using SMS reported no improvement in polio vaccination [12]. Factors such as health literacy and gender difference in the response to interventions could potentially influence their effectiveness [11,13].

### Research Gap

Although numerous studies have been conducted to assess the potential of using mHealth in vaccination programs, to the best of our knowledge, there is no comprehensive review that has assessed the effectiveness of mHealth interventions targeting parents and children worldwide to address the HPV vaccination gap. The current limitations in our understanding of how mHealth could affect HPV vaccination intentions among unvaccinated, US children and their parents and its impact on actual vaccine uptake highlight an urgent need for studies in this area to better inform and refine the intervention strategies.

### Objective

This systematic review was conducted to describe, assess, and synthesize the effectiveness of mHealth interventions in promoting HPV-related knowledge, vaccination intentions, and behaviors (uptake) among unvaccinated children and their parents. In the context of this study, “effectiveness” was measured based on the intended outcomes of the intervention, which included promoting HPV-related knowledge and increasing the vaccination intention or actual vaccine uptake among parents or youth. The findings will inform directions for developing effective mHealth interventions to mitigate HPV-related cancers through vaccination.

## Methods

### Study Design

**Figure 1:**
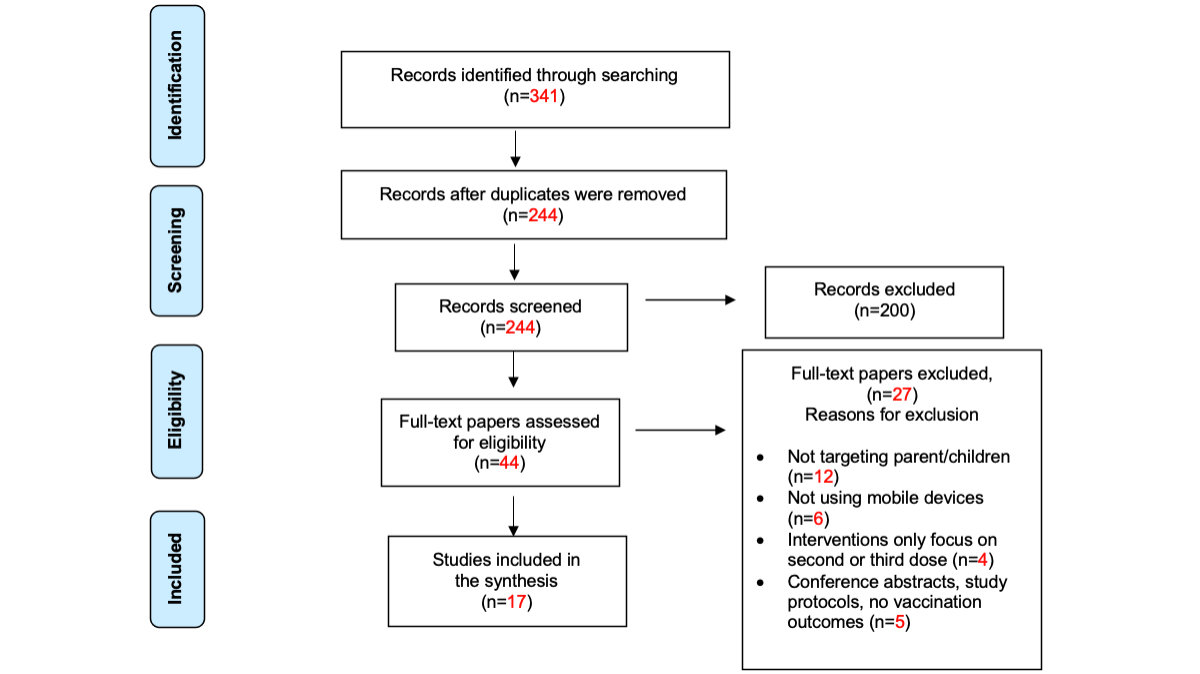
PRISMA (Preferred Reporting Items for Systematic Reviews and Meta-Analyses) flow diagram of the study selection process.

The authors of this paper conducted a comprehensive literature search and gathered and appraised empirical evidence from databases including Google Scholar, PubMed, CINAHL, PsycINFO, and Cochrane Library. Specifically, the PRISMA (Preferred Reporting Items for Systematic Reviews and Meta-Analyses) statement guided this review process. The PRISMA flow diagram (Figure 1) illustrates the study selection process, reasons for study exclusion, and number of papers obtained and retained. Refer to Multimedia Appendix 1 for the PRISMA checklist.

### Inclusion Criteria

Inclusion criteria were as follows: (1) peer-reviewed studies published in English from January 2011 to December 2022; (2) mHealth interventions aimed to improve youth’s HPV vaccination rates; (3) targeted parents of unvaccinated youth or unvaccinated youth alone; and (4) measured HPV-related knowledge, HPV vaccination intention, or vaccine uptake. Conference abstracts, letters to the editor, and study protocols were excluded.

### Search Strategy

Overall, 3 researchers independently conducted the review and identified potential eligible studies. The title, abstract, and full text of the studies were assessed for inclusion. The reference lists of the included studies were also screened. Medical Subject Heading terms and keywords of the potential eligible papers were both used for the search. Words used for the search were: (“Mobile health” or “mHealth” “eHealth” or “mobile application”), (“Human Papillomavirus” or “HPV”), (“Human Papillomavirus vaccination” “Human Papillomavirus intervention” or “HPV vaccination” or “HPV intervention), (“HPV vaccination intention” or “HPV vaccine uptake”), (“parents” or “caregivers” or “mother” or “father”), and (“children” or “adolescent” or “youth” or “teenager”). Searching strategies for searched databases are presented in Multimedia Appendix 2.

### Evidence Assessment and Synthesis

The level of evidence of the included studies was assessed using the modified Melnyk Levels of Evidence [14]. In addition, each study was assessed for its risk of bias and level of certainty in evidence using the Cochrane Grading of Recommendations Assessment, Development, and Evaluation methodology [15]. The risk of bias was categorized into 3 levels: high, low, or unclear. These were determined based on limitations in the body of evidence, which could subsequently downgrade the quality of evidence from high to very low. Disagreements among the 3 researchers were resolved with discussions until 100% consensus was achieved. Owing to heterogeneity across the studies and variations of evaluated outcomes, a meta-analysis was not performed for the included studies. Thus, we synthesized the evidence by analyzing and comparing the characteristics of these studies and the effectiveness of the mHealth interventions on youth’s HPV vaccination intentions and vaccine uptake and discussed the factors associated with the intervention impacts. Specifically, a narrative approach was used, in which the results from individual studies were described and discussed. Details of the studies, intervention characteristics, and findings of each included study are presented in table format to facilitate a clear visual assessment. Studies were grouped according to factors including the study location, design, types of the intervention, outcomes measured, evidence level, and quality of evidence.

### Ethical Considerations

Ethics approval was not required for this systematic review as it involves the analysis of previously published data. No new primary data were collected or used for this study.

## Results

### Included Studies

The search initially obtained 341 papers, of which 97 (28.4%) were duplicates. After removing the duplicates, the remaining 71.6% (244/341) of the papers were further screened. The primary reason for exclusion at the screening stage was that the study did not focus on HPV vaccination (141/341, 41.3%) or mHealth interventions (59/341, 17.3%). Overall, 44 papers were included for the subsequent full-text review, and 27 (61%) papers were excluded with reasons after assessing the eligibility. Specific reasons for study exclusion included the following: mHealth interventions not targeting parents or children (12/27, 44%); interventions not using mobile devices (6/27, 22%); interventions only focused on the second or third dose of the HPV vaccine uptake (4/27, 15%); and conference abstracts, study protocols, or studies that did not measure vaccination outcomes (5/27, 19%). Overall, 39% (17/44) of the papers were included in the final review.

### Study Characteristics

The characteristics of the 17 included studies are described in Table 1. Most studies (14/17, 82%) included in the review were conducted in the United States [16-29]. The remaining 18% (3/17) of the studies were conducted in the Netherlands, Australia, and Japan, respectively [30-32]. Regarding study settings, 59% (10/17) of the studies recruited the study participants from clinical settings including primary care practice, public health clinics, vaccine clinics, family medicine, pediatric and adolescent clinics, and outpatient clinics [16-19,21,24,25,27-29]. Of the 17 studies, 2 (12%) used both clinic and community recruitment [22,23]; 1 (6%) collaborated with secondary schools to implement the intervention [31]; and 4 (24%) relied on web-based outlets for participant enrollment, with 2 (12%) referring to the National Immunization Register and Surveys [26,30] and 2 (12%) using designated web pages [20,32].

Regarding the target population, of the 17 studies, 9 (53%) comprised diverse racial and ethnic participants [16,19-21,23,25,26,28,29]; 5 (29%) specifically focused on 1 racial and ethnic group, such as African Americans, Vietnamese Americans, Asian participants, or participants of Mexican heritage [17,18,22,24,32]; and 3 (18%) did not specify the racial and ethnic groups they included [27,30,31]. Furthermore, of the 17 studies, 13 (76%) targeted parents [16,18,19,22-32], with 15% (2/13) of them specially focused on mothers [26,30]. A study solely included youths aged 12 years [30], and 76% (13/17) of the other studies involved adolescents aged between 10 and 18 years [16-21,24-29,31]. For the 41% (7/17) of the studies that reported information about the parents’ ages, the average age spanned from 38 to 48 years [18,22,23,26,28,29,32].

Among the 17 studies, initiation of at least 1 dose of HPV vaccine was measured as the primary outcome in 12 (71%) interventions [16-21,24,25,27,29-31], and 3 (18%) of the studies also assessed the completion of the vaccine series [17,21,27]. The intention of parents to vaccinate their children against the virus, which was found in 35% (6/17) of the studies, was the second most frequently measured outcome [22-24,26,28,32]. Apart from the 35% (6/17) of studies that examined HPV-related knowledge [18,21-23,28,29], 29% (5/17) of studies also evaluated other determinants of the vaccination, such as attitudes and beliefs toward the vaccine, normative beliefs, facilitators of and barriers to vaccination, perceived susceptibility, and severity of the infection [17,18,22,23,30]. Regarding the study design and quality of the design, of the 17 studies, 12 (71%) used randomized controlled trials [16,17,19,21-27,29-32], 4 (24%) were quasi-experiments [18,20,22,23], and 1 (6%) used a mixed methods approach [28].

Following the Melnyk Levels of Evidence approach, among the 17 studies, 13 (76%) were of level-2 evidence including the mixed methods study [16,17,19,21,24-32] and 4 (24%) quasi-experimental studies [18,20,22,23] were of level-3 evidence [14]. The quantitative elements contained in the mixed methods study were used to determine the level of evidence. Moreover, of the 17 studies, 13 (76%) were found to be of moderate risk of bias [16,18-23,25,26,28-30,32] and 4 (24%) were of high risk [17,24,27,31]. The downgrade in evidence quality was owing to inadequate descriptions of allocation concealment, blinding procedures, and psychometrics of outcome measures.

**Table 1:** Summary of studies included in the review.

Study, year	Location	Design	Intervention	Outcome measures	Evidence level	Quality of evidence
Rand et al, 2015 [16]	New York, United States	Randomized controlled trial	Centralized Managed Care Organization–generated text message reminders	The receipt of the first and subsequent doses of the HPV^a^ vaccine	2	Moderate
DiClemente et al, 2015 [17]	Atlanta, United States	Randomized controlled trial	Computer-delivered presentation and motivational keychains with vaccine reminders	Perceived susceptibility and severity of contracting HPV and initial HPV vaccine uptake and dosage compliance	2	High
Chen et al, 2017 [18]	Arizona, United States	Quasi-experimental design	Bilingual, tablet-tailored intervention	HPV-related knowledge, cultural norms, facilitators of and barriers to HPV vaccination, parental intention to vaccinate their children, and the receipt of the first dose of vaccine	3	Moderate
Pot et al, 2017 [30]	Dutch	Randomized controlled trial	Website with web-based assistants	HPV vaccination uptake, mothers’ degree of informed decision-making, decisional conflict, and critical determinants of vaccination uptake	2	Moderate
Hofstetter et al, 2017 [19]	New York, United States	Randomized controlled trial	Automated text reminders with educational messages	The receipt of the first dose of HPV vaccine and missed vaccination opportunity by 4, 12, and 24 wk after the initial reminder	2	Moderate
Mohanty et al, 2018 [20]	Philadelphia, United States	Quasi-experimental design	Facebook vaccination campaign	The receipt of the first, second, and third doses of the HPV vaccine through the Facebook page, 3 for ME	3	Moderate
Ortiz et al, 2018 [21]	Southeastern Cities, United States	Randomized controlled trial	Social media health intervention	Knowledge related to HPV and its vaccine, interpersonal discussions about HPV and its vaccine, and HPV vaccine immunization status	2	Moderate
Tull et al, 2018 [31]	Victoria, Australia	Randomized controlled trial	Motivational and self-regulatory SMS	The receipt of the HPV vaccine (any dose) and completion of the HPV vaccine schedule	2	High
Chen et al, 2019 [22]	Arizona, United States	Quasi-experimental design	Tablet-tailored intervention	HPV-related knowledge, perceived risk of having HPV, facilitators of and barriers to HPV vaccination, cultural norms, parental intention to vaccinate their children, and the receipt of the first dose of vaccine	3	Moderate
Chen et al, 2019 [23]	Arizona, United States	Quasi-experimental design	Digital storytelling	HPV-related knowledge, attitudes, and beliefs and mothers’ intent to vaccinate their children against HPV	3	Moderate
Dempsey et al, 2019 [24]	Colorado, United States	Randomized controlled trial	Web-based, individually customizable intervention	The HPV vaccination intention among parents, receipt of any needed dose of the HPV vaccine, and initiation and completion of the vaccine series	2	High
Dixon et al, 2019 [25]	Indiana, United States	Randomized controlled trial	Tablet-based educational intervention	The changes in HPV vaccine status, including the first dose (initiation), second dose, and third dose (completion) of the vaccine series	2	Moderate
Panozzo et al, 2020 [26]	27 states in the United States with the lowest HPV vaccine coverage	Randomized controlled trial	Web-based digital videos with tailored messages	Mothers’ intent to vaccinate their children and the changes in the strength of the main HPV vaccine concern	2	Moderate
Szilagyi et al, 2020 [27]	New York, United States	Randomized controlled trial	Auto dialer centralized reminder and recall	The initiation and completion of the HPV second or third dose of the vaccine series	2	High
Suzuki et al, 2021 [32]	Tokyo, Japan	Randomized controlled trial	Web-based educational intervention	HPV awareness, attitudes toward the HPV vaccination, and willingness of adults to consider children’s vaccination	2	Moderate
Becker et al, 2022 [28]	Texas, United States	Mixed methods study	Smartphone app	Awareness, attitudes, and knowledge regarding HPV and the vaccine; intentions to vaccinate their child; and communication with the child’s pediatrician about the vaccine	2	Moderate
Shegog et al, 2022 [29]	Texas, United States	Randomized controlled trial	Smartphone app	Knowledge about HPV and the HPV vaccine and HPV vaccination initiation rates	2	Moderate

^a^HPV: human papillomavirus.

### Intervention Characteristics

This review encompassed a range of mHealth interventions. Of the 17 studies, 5 (29%) examined the impact of tailored educational information regarding the HPV vaccine, with 60% (3/5) of them using web-based interventions to deliver the messages [24,30,32] and 40% (2/5) using tablet computers [18,23]. Messaging services were used in 24% (4/17) of the studies [16,19,27,31], 75% (3/4) of which featured SMS text messages with embedded educational and customized content [19,27,31]. Digital videos were used in 18% (3/17) of the studies, 67% (2/3) of which incorporated customized messages to address the vaccination concerns of parents or guardians [25,26], whereas the other study used personal stories from mothers of vaccinated adolescents sharing their experiences with HPV and the vaccine [22]. In 12% (2/17) of the studies, Facebook pages were used to reach the targeted adolescents for the intervention [20,21]. Of the 17 studies, 2 (12%) studies used smartphone apps to support parents in making decisions regarding HPV vaccination [28,29]. A study used the combination of a computer-delivered presentation on HPV vaccination, vaccine appointment cards, and keychains with motivational health messages [17].

In addition, the interventions in 18% (3/17) of the studies incorporated culturally tailored and gender-tailored messages addressing cultural beliefs pertinent to HPV vaccination [17,18,22]. Of the 17 studies, 4 (24%) indicated that the development of their interventions was informed by multiple sources. These included key informants; significant community and end user input; and insights from adolescents, parents, and health care providers. This comprehensive approach aimed to capture each individual’s unique questions, experiences, attitudes, and beliefs [21-24].

The duration of the intervention was reported in 35% (6/17) of the studies, and it varied depending on the type of intervention, ranging from <50 seconds to 30 minutes [17,18,22,23,25,26]. The delivery frequency of message reminders also varied, with some interventions providing reminders once [31], and others providing up to 4 [16] or 5 weekly messages [19]. However, 18% (3/17) of the studies did not specify the duration of their interventions [24,30,32]. In 18% (3/17) of the studies, only the total duration of the study period was reported, with 2 Facebook-based vaccination campaigns lasting 1 year and 3 months, respectively [20,21], and a study exploring the use of phone reminders lasting 2 years [27]. Another 12% (2/17) of the studies used apps that were assessed by logging data over 5 months [28,29].

Among the 17 studies, 10 (59%) incorporated theories into their interventions. Theories of behavior change were used in 53% (9/17) of the studies to guide the development of the interventions [17,18,20-23,27-29,32], including the Information-Motivation-Behavioral Skills model, PRECEDE-PROCEED Model, Health Belief Model, Theory of Planned Behavior, Social Cognitive Theory, and Theory of Reasoned Action. A study of digital storytelling used the Model of Culture-Centric Narratives in Health Promotion as the guiding theoretical framework [22].

### Measured Outcomes

#### HPV-Associated Knowledge, Attitudes, and Beliefs

Of the 17 studies, 6 (35%) that used educational interventions found that the interventions positively influenced HPV-related knowledge, with knowledge scores showing improvement after the intervention [18,21-23,28,29]. The knowledge was assessed through various measures, specifically covering topics including the recognized risk factors for HPV infection, associated diseases, and methods for HPV detection [18]; information about how HPV is sexually transmitted, its symptoms, prevalence, potential to lead to cancer and genital warts in both sexes, and vaccination requirements for both boys and girls [21]; notion of selective vaccination of boys and girls [22]; and HPV risks in teenagers, its ties to different diseases and cancers, preventive approaches, recommended vaccine doses, and its safety [23]. A study assessed different health conditions that the HPV vaccine could prevent and people at risk for HPV infection [29], and another study solely measured the increase in knowledge about HPV and its vaccine using a 4-point agreement scale [28]. In several papers (4/17, 24%) [22,28,29,32], favorable changes in belief and attitude toward HPV vaccination and its safety and effectiveness were also found among parents who had been enrolled in and completed the intervention.

#### Facilitators of and Barriers to HPV Vaccination

Among the 17 studies analyzed, 2 (12%) [18,23] noted a significant rise in parents' perception of facilitators for HPV vaccination between the preintervention and postintervention phases, with *P* values of .008 and .007, respectively. Several factors facilitating vaccination behavior change were identified and assessed regarding the intervention effectiveness, such as suggestions or recommendations from health care providers or religious leaders, positive attitudes toward the vaccine, support from significant others, and beliefs about vaccine benefits.

In the 12% (2/17) of studies mentioned previously, perceived barriers to vaccination were also compared before and after the intervention. Nevertheless, there was no statistically significant association identified. Factors examined included concern about vaccine safety, lack of health insurance or provider recommendation, language barrier between patients and providers, and worries that the vaccination would encourage early sexual activity.

Moreover, in a study [17], perceived susceptibility or severity of HPV and perceived risk of HPV developing into cervical cancer served as motivating factors for HPV vaccination. The findings indicated that participants exposed to the media intervention had a high level of perceived susceptibility or severity of HPV and HPV‐attributable cancers compared with those in the control group. The difference in perceived susceptibility between the 2 study conditions was statistically significant (*P=*.03).

#### Cultural Norms or Beliefs

Of the 17 studies, 4 (24%) evaluated culturally congruent interventions [17,18,22,23], whereas only 2 (12%) of them specifically included cultural beliefs or cultural norms in the outcome measures [18,23]. Despite the computer-tailored intervention using information from sources such as focus groups with parents and health care providers and targeting HPV knowledge and awareness among the youth, the results found no significant difference in cultural norms among parents with unvaccinated adolescents before and after the intervention [23]. However, significantly decreased scores of vaccination-related cultural beliefs (*P*<.001) and more favorable attitudes toward the vaccine were identified among Mexican-heritage participants in the study [18].

#### Parental Intention and Vaccine Decision-Making

Of the 17 studies, 8 (47%) [18,22-24,26,28,30,32] evaluated changes in participants’ intention or willingness to vaccinate after the intervention. Of the 8 studies, 3 (38%) [18,22,23] revealed that the parents’ intention to vaccinate their youth was ranging from 95% to 100% during the postintervention period. Of the remaining studies, 63% (5/8) included at least one comparison group. All of these (5/5, 100%) demonstrated a positive intervention impact, showing a significantly higher intention to vaccinate children compared with the control group, with *P* values ranging from .002 to <.001. A study [30] found that the tailored intervention, where messages were customized to mother participants’ preferences and needs for HPV vaccine decision-making, was particularly useful for them when in doubt, as they experienced less decisional conflict relating to a lack of information after the intervention. However, a study [26] found that the average strength of the main concern about the HPV vaccine among mothers remained high even after receiving the intervention messages tailored to reduce the concerns.

#### Vaccine Uptake and Completion

Of the 41% (7/17) studies discussing mHealth's impact on youth's HPV vaccine initiation and completion of HPV vaccine, all tracked this over time, offering data up to 7 months after intervention [16,20,21,24,25,27,31]. Overall, 29% (5/17) of the studies [16-18,25,31] reported notable increases in HPV vaccination rates and vaccine series completion rates. Of these 5 studies, 3 (60%) assessed youth participants’ receipt of the initial HPV vaccine dose [16,18,25] and 2 (40%) evaluated youth participants’ adherence to the second and third doses of the HPV vaccine series [17,31]. Study [19] noted that after 12 weeks of plain text reminders, there was a significant increase in vaccine uptake among those aged 11 to 12 years (*P*=.07), but this was not observed in the 13 to 17 age group.

In contrast, the findings of 29% (5/17) of the studies suggested no difference in youth vaccine uptake between the intervention and control groups. Of the 17 studies, 3 (18%) [21,27,30] found no significant difference in the vaccination uptake and completion rates across the intervention and control groups. A study [24] found that the HPV vaccine uptake among Latino adolescents and young adults did not differ significantly across the groups receiving tailored educational messages, nontailored interventions, and usual care despite their improved vaccination intention. The Facebook-based vaccination campaign successfully reached and engaged >12,000 adolescents, whereas only 73 of them received the first dose of the vaccine [20].

#### Intervention Engagement

In 24% (4/17) of the studies that evaluated intervention fidelity and engagement, several methods were used. For a smartphone app, qualitative feedback was collected and revealed that participants preferred push notifications for HPV facts and suggested that the app could incorporate broad topics, be more interactive, and contain adolescent-friendly elements such as animations and games [28]. User interactions, such as clicks, likes, comments, and shares, on a vaccine campaign Facebook page were analyzed to determine whether youth participants preferred advertisements highlighting immediate risks (eg, HPV-related infections) as opposed to long-term consequences (eg, HPV-induced cancer). The findings suggested that youth participants might be more responsive to content that focuses on immediate risks; however, further analysis was not conducted to identify how this reception might influence vaccination intent and uptake [20]. Another study of educational messaging intervention proposed an approach to assess the time users engaged with web-based intervention pages to evaluate their involvement, with results still under analysis [24]. Moreover, a review was conducted to assess both recall and intervention fidelity, where participants were asked to identify which notifications and specific pieces of information from the Facebook-based intervention they remembered. However, there was no improvement in HPV knowledge or vaccination rates among those using the intervention [21].

## Discussion

### Summary

As of 2022, the HPV vaccination has been introduced in >120 countries worldwide; however, challenges for youth vaccination remain [33]. Digital technologies such as mHealth could be applied to address this global public health concern. This systematic review explored the effectiveness of 17 mHealth interventions in improving the HPV vaccination among unvaccinated youth. The included mHealth interventions used various technologies, including SMS text message reminders, smartphone apps, computer or web-based tailored interventions, social media campaigns, digital videos, and digital storytelling. The findings of the review suggested that mHealth intervention was a promising approach to improving the low HPV vaccination rates among youth. Specifically, these mHealth interventions could effectively enhance knowledge, attitudes, and beliefs related to HPV and its vaccine and motivating factors including perceived susceptibility or severity of HPV infections and its related cancer, which in turn, assisted in making vaccination decisions and promoting the vaccine uptake.

Tailored interventions, which considered the unique needs and concerns of specific groups or individuals, were developed using theoretical components and messaging that resonated with participants in a context-specific manner, such as through cues related to age, gender, race and ethnicity, or culture. These efforts made them more effective in addressing facilitators of and barriers to vaccination and eliciting positive responses for health behavior change compared with the information-only approach [34]. Consistent with previous studies, we found that tailored messages within successful mHealth interventions in this review addressed the knowledge gaps regarding HPV and its vaccine. The customized educational messages were designed with consideration of a variety of factors, including previous empirical evidence; theoretical concepts in the guided framework (eg, knowledge, attitudes, beliefs, perceived risk, barriers, and facilitators); age, gender, ethnicity, race, and cultural beliefs of the children regarding HPV vaccination; youth’s current knowledge about the disease and vaccine based on assessment results; feedback from parents in the clinic network obtained through focus groups; inputs from youth advisory board meetings; and insights from other important sources such as health care providers and religious leaders [18,21-23,28,29].

Although it is noteworthy that tailored information is crucial for enhancing the uptake of HPV vaccination among unvaccinated youth and their parents, tailored education that addressed all the concerns of parents was found to be more effective in improving their intentions to vaccinate children than focusing solely on their primary concerns [26]. The results aligned with those of previous studies indicating that parents who had vaccine hesitancy were more likely to refuse the HPV vaccine owing to a range of concerns related to the importance of the vaccine for both the child and community health, vaccine safety, potential side effects, and overall benefits [35]. Boosting parents’ confidence in the HPV vaccine by addressing their concerns and discussing the benefits of the vaccination could lead to a reduction in their hesitancy toward the vaccine [36].

Cultural norm was also found to be associated with the HPV vaccine administration. Parental and youth reluctance to seek the HPV vaccine may be influenced by their traditional beliefs, such as cultural norms that advocate sexual abstinence until marriage [37]. A previous study conducted among ethnic minority parents revealed concerns about the vaccine, stemming from fears that it might encourage risky sexual behavior and promiscuity [38]. Although this study was specifically centered on the ethnic minority population, similar views could also be prevalent among other groups that upheld beliefs valuing a woman’s purity and reputation, especially in sexual or relational contexts [39]. These attitudes, in turn, could influence the perceptions about and use of health care services and their self-reported health, health behaviors, and health outcomes [40,41]. Nevertheless, providing relevant and reliable information about the HPV vaccine to parents could help to drive positive change in their attitudes toward the vaccination [18,23,37]. Both religious beliefs and cultural views may influence vaccine decision-making [37]. Hence, engaging spiritual organizations and religious authorities in the design of interventions aimed at increasing HPV vaccination rates among unvaccinated youth could be essential.

Of the 17 studies, 3 (18%) [20,21,30] found that web-based interventions or interventions using social media had limited effects on increasing the actual uptake of the HPV vaccine, despite improved knowledge about the virus and vaccine and vaccination intention among the participants. Although social media platforms such as YouTube, Facebook, Instagram, and Twitter could be a feasible and effective tool with expansive reach and be useful for disseminating health information about the vaccine, providing practical information about how and where to receive the vaccine, and addressing public shared concerns or questions, these interventions may not be sufficient on their own to promote vaccination behavior change among adolescents [42]. The information that individuals receive through social media is not always complete or favorable. To gain a deep understanding of how unvaccinated youth and their parents perceive and trust the information, it is important to explore the factors that influence strategic messaging. This could provide valuable insights into what types of information could be useful and credible for this population.

Although the use of SMS text message interventions had been shown to have a small impact on vaccine uptake and completion among eligible adolescents, positive outcomes varied among states and regions even when using identical messages, regardless of the number of messages sent [27]. These message reminders may only serve as an additional stimulus for those who have already decided to vaccinate [19]. Factors such as timing and setting for the message sent and easy access to vaccination may also be critical to consider in reducing the vaccination gap. To improve youth attendance and stay on track to complete the vaccine schedule, integrating SMS reminders with vaccine appointment alerts could be beneficial. In addition, incorporating secondary school–based vaccination programs, where parents were provided with consent cards and informational booklets and were reminded of upcoming vaccination sessions through school newsletters or a web-based portal, could further increase vaccine completion rates for the second and third doses in the HPV vaccine series [31]. These findings were in accordance with previous studies showing that vaccination interventions were more effective when framed as reminders to receive the vaccine that had already been prescheduled for the message receivers. Moreover, SMS text messages integrated with behavior change theories and sent before health care visits had the potential to significantly boost vaccination rates (*P*<.05) [43]. The effectiveness of messaging interventions and the direction of the effect may vary based on context. Further studies are needed to determine how successful SMS text message interventions could be adapted across subpopulations and different settings.

The improved HPV vaccination intention among parents may not necessarily translate well into youth HPV vaccine use as suggested by Dempsey et al [24]. Involving youth in vaccination decision-making and addressing their needs in the process may help them take responsibility for their health. In addition, adolescents aged as young as 9 to 12 years may already have the capability to make informed medical decisions [44]. Nonetheless, the review revealed that there was a lack of mHealth interventions that targeted unvaccinated adolescents, with only 4 studies focused on this population. The understanding of successful interventions that could help improve youth vaccination rates could be limited owing to the lack of research in the area. Further studies that investigate adolescent vaccination decision-making and its changes before and after interventions could provide valuable insights into the factors that contribute to effective interventions. These insights could then be used to facilitate the translation of improved vaccination intentions into actual increases in vaccination rates among youth.

Other aspects such as participants’ satisfaction and experience with the intervention (utility, usefulness, and quality of the mobile app) were critical to assess [28]. In 24% (4/17) of the studies, the acceptability of the interventions by parents and youth was evaluated. This assessment addressed various aspects of the interventions, including the appropriateness of content and wording, graphic design and color choices, intervention length, the likelihood of recommending the intervention, and discussions about the intervention [18,21,23,30]. These evaluations allowed for a comprehensive assessment of the intervention and provided areas for ongoing improvements to the technology-mediated, tailored intervention for the HPV vaccination. The findings may also help to inform how to link intervention engagement to youth vaccination and uncover motivational factors that could influence significant vaccination behavior change and adherence.

This systematic review expands the current understanding regarding the use of mHealth interventions and their impact on unvaccinated youth and their parents. It adopted multiple search strategies and was not limited to randomized controlled trials; such inclusion could help gain a more comprehensive understanding of the strengths and limitations of the available evidence. Although this review could be a useful reference to guide the future development and evaluation of mHealth interventions targeting the improvement of HPV vaccination decisions and behaviors among youth, it is essential to note that the studies of moderate quality included in the review may have the potential to compromise the generalizability and transferability of the findings.

### Limitations

The review also has some limitations, including the limited number of databases searched; exclusion of non–English language studies; and a narrow geographical focus, with most studies conducted in the United States, which could all lead to selection and reporting biases. The evidence presented in the review could not represent all the current mHealth interventions for HPV vaccination as only parents and adolescents were targeted. Moreover, a meta-analysis could not be conducted because of the heterogeneity of the studies and measured outcomes. Finally, the dynamics surrounding HPV vaccine decision-making might be different in various regions outside the United States. For instance, the accessibility or availability of the HPV vaccine might be a predominant factor affecting vaccine decisions in some other countries or regions [45,46]. Although this study sheds light on the situation within the United States, further studies are necessary to explore the nuanced interactions between mHealth tools and vaccine decision-making in diverse international settings.

### Conclusions

This review synthesized the available evidence for mHealth interventions designed to promote HPV vaccination among youth. mHealth has shown promising results in improving youth and their parents’ HPV-related knowledge, attitudes, and beliefs toward the vaccine and addressing the barriers to vaccination, vaccination intention, and vaccine uptake. Future studies should examine the factors that could increase the quality of the study evidence and the complexity of the use of mHealth vaccination interventions, as its impact could differ depending on the platforms used, populations involved, settings of the app, and any additional efforts used to encourage the vaccination.

## Appendix

Multimedia Appendix 1 PRISMA (Preferred Reporting Items for Systematic Reviews and Meta-Analyses) checklist.

Multimedia Appendix 2 Searching strategies for searched databases.

## Abbreviations

HPV: human papillomavirus
mHealth: mobile health
PRISMA: Preferred Reporting Items for Systematic Reviews and Meta-Analyses
