# Supplementary material for: The Effectiveness of mHealth Interventions Targeting Parents and Youth in Human Papillomavirus Vaccination: Systematic Review
Source: JMIR Pediatr Parent. 2023 Nov 21;6:e47334. doi: 10.2196/47334 (PMC10698656; doi:10.2196/47334)
Supplement: Multimedia Appendix 2 [file pediatrics_v6i1e47334_app2.pdf]

Appendix 2: Searching strategies for searched databases.

Databases	Searching Terms	Number of Studies	Searching Period
Google Scholar	"mHealth" and "mobile health" and "eHealth" and "parent" and "children" and "adolescent" and "Human Papillomavirus" and "HPV" and "Vaccine"	240	From January 2011 to December 2022
PubMed	("Mobile health"[MeSH Terms] OR "mHealth" [MeSH Terms] OR "eHealth" [MeSH Terms] OR " mobile application" [All Fields]) AND ("Human papillomavirus"[MeSH Terms] OR " Human Papillomavirus vaccines" [MeSH Terms] OR "HPV vaccines" [MeSH Terms] OR "Human Papilloma virus vaccine" [MeSH Terms] [All Fields]) AND ("vaccination"[MeSH Terms] OR" vaccine uptake" OR "vaccination intention" [All Fields]) AND ("adolescent"[MeSH Terms] OR "child" [MeSH Terms] OR "teenager" OR "youth" [All Fields]) AND ("parents" "[MeSH Terms] OR "legal guardians"[MeSH Terms] OR "mothers" [MeSH Terms] OR "fathers" "[MeSH Terms] OR "caregiver " [MeSH Terms] [All Fields])	85	
CINAHL	"mhealth" or "mobile health" or "m-health" or "mobile app" or "mobile application" AND "hpv vaccine" or "hpv vaccination" or "human papillomavirus vaccine" AND "adolescents" or "teenagers" or "teen" or "youth" AND "parents" or "caregivers" or "mother" or "father"	8	
PsycINFO	"mobile health" or "mhealth" or "mobile app" or "mobile application" AND "hpv vaccine" or "hpv vaccination" or "human papillomavirus vaccine" AND "adolescents" or "teenagers" or "teen" or "youth" AND "parents" or "caregivers" or "mother" or "father"	4	

Cochrane Library	"mobile health" or "mhealth" or "mobile app" or "mobile application" AND "human papillomavirus" or "hpv" AND "human papillomavirus vaccine" or "hpv vaccination" or "vaccine uptake" or "vaccination intention" AND "adolescents" or "teenagers" or "teen" or "youth" AND "parents" or "caregivers" or "mother" or "father" or "legal guardians"	4	
Total searched articles		341	
Total articles that fulfill the eligibility criteria for review		17	
